# Evolutionary insights of *Bean common mosaic necrosis virus* and *Cowpea aphid-borne mosaic virus*

**DOI:** 10.7717/peerj.6297

**Published:** 2019-02-13

**Authors:** James M. Wainaina, Laura Kubatko, Jagger Harvey, Elijah Ateka, Timothy Makori, David Karanja, Laura M. Boykin, Monica A. Kehoe

**Affiliations:** 1 School of Molecular Sciences and Australian Research Council Centre of Excellence in Plant Energy Biology, The University of Western Australia, Crawley, Western Australia, Australia; 2 Ohio State University, Columbus, OH, United States of America; 3 Feed the Future Innovation Lab for the Reduction of Post-Harvest Loss, Kansas State University, Manhattan, KS, United States of America; 4 Department of Horticulture, Jomo Kenyatta University of Agriculture and Technology, Nairobi, Kenya; 5 Kenya Agricultural and Livestock Research Organization (KARLO), Machakos, Kenya; 6 Plant Pathology, Department of Primary Industries and Regional Development Diagnostic Laboratory Service, South Perth, Australia

**Keywords:** Phylogenomics of BCMNV and CABMV, Virus evolution, Genomics, Next-generation sequencing (NGS), Kenya, Smallholder farmer

## Abstract

Plant viral diseases are one of the major limitations in legume production within sub-Saharan Africa (SSA), as they account for up to 100% in production losses within smallholder farms. In this study, field surveys were conducted in the western highlands of Kenya with viral symptomatic leaf samples collected. Subsequently, next-generation sequencing was carried out to gain insights into the molecular evolution and evolutionary relationships of Bean common mosaic necrosis virus (BCMNV) and Cowpea aphid-borne mosaic virus (CABMV) present within symptomatic common bean and cowpea. Eleven near-complete genomes of BCMNV and two for CABMV were obtained from western Kenya. Bayesian phylogenomic analysis and tests for differential selection pressure within sites and across tree branches of the viral genomes were carried out. Three well–supported clades in BCMNV and one supported clade for CABMNV were resolved and in agreement with individual gene trees. Selection pressure analysis within sites and across phylogenetic branches suggested both viruses were evolving independently, but under strong purifying selection, with a slow evolutionary rate. These findings provide valuable insights on the evolution of BCMNV and CABMV genomes and their relationship to other viral genomes globally. The results will contribute greatly to the knowledge gap involving the phylogenomic relationship of these viruses, particularly for CABMV, for which there are few genome sequences available, and inform the current breeding efforts towards resistance for BCMNV and CABMV.

## Introduction

Legumes and in particular common bean (*Phaseolus vulgaris L.*) and cowpea (*Vigna unguiculata L.*) are the primary source of proteins in developing countries and in particular within the sub-Saharan Africa (SSA) region. However, one of the major limitations in legume production are viruses, among them are bean common mosaic necrosis virus and (BCMNV) cowpea aphid-borne mosaic virus (CABMV), both of which belong to the genus *Potyvirus* (Li et al., 2014; Worrall et al., 2015). These viruses are some of the most economically important viruses affecting *Fabaceae* (*Leguminosae*) globally, and have a wide host range and are reported in both cultivated and native vegetation (Li et al., 2014; Worrall et al., 2015). Production losses attributed to BCMNV are up to 100% whereas for CABMV they range from 30–60% (Damayanti et al., 2008; Damiri, Abdalla & Amer, 2013; Mangeni et al., 2014). The modes of transmission of these viruses are via infected seed stocks as well as through viruliferous aphids (Worrall et al., 2015).

There have been no studies in Kenya on the molecular evolution of BCMNV and CABMV, particularly within the western highlands of Kenya. A majority of studies in this region are based on serological assays and biological characterisations (Mangeni et al., 2014). Beans and cowpea play an important role as part of an intercrop in a predominantly mixed cropping system among smallholder farmers in Africa, and their role in human nutrition cannot be underestimated. Firstly, they are a source of proteins and micronutrients (iron) especially for women and children in low-income households. Secondly, over the last decade, there has been intensive advocacy in the use of legumes as intercrops with cereals such as maize, sorghum, millet and cassava, which are prioritized as essential crops both in Kenya and in the larger SSA region. This has resulted in enhanced interregional seed trade between smallholder farmers and within neighboring countries (Uganda and Tanzania), with minimal phytosanitary regulation (Katungi et al., 2009). In addition, legumes are currently utilised for integrated pest management of whitefly and whitefly-transmitted viruses (Fondong et al., 2000; Lapidot et al., 2014; El-sawy, Mohamed & Elsharkawy, 2014; Uzokwe et al., 2016).

BCMNV and CABMV are monopartite, single-stranded positive-sense RNA viruses with an average genome size of ∼10 Kb and a polyA tail on the 3′-terminal end (Fang et al., 1995; Mlotshwa et al., 2002; El-sawy, Mohamed & Elsharkawy, 2014; Kehoe et al., 2014a). They encode a single polyprotein that is processed into ten proteins in a similar order and function to all members of the family *Potyviridae:* P1 (Symptomatology), HcPro (Aphid transmission), P3 (pathogenicity), 6K1, CI (ATPase/RNA helicase, cell to cell movement), 6K2 (Anchoring the viral replication complex to membranes), Nla-Vpg, Nla-Pro Nlb (Genome replication, RNA-dependent RNA polymerase (RdRP) and coat protein (CP) (Aphid transmission, virus assembly, cell to cell and systemic movement). There is also a second open reading frame called the PIPO (virulence dependency) (Urcuqui-inchima & Haenni, 2001; Chare & Holmes, 2004; Cui et al., 2017). The small nature of the viral genome and the broad range of environments in which they circulate serve as strong drivers of their genome evolution (Chare & Holmes, 2004).

RNA viruses undergo multiple mutations during the replication phase; thus their evolutionary rates (sub/site/year) are considerably higher than cellular genes within their host (Chare & Holmes, 2006; Duffy, Shackelton & Holmes, 2008; Holmes, 2009). These nucleotide mutations may result in no change to the amino acids (synonymous changes per synonymous sites (dS)), or a change in the amino acid (non-synonymous changes per non-synonymous sites (dN)). The relative magnitude of these types of changes can lead to positive selection (dN/dS > 1), purifying selection (dN/dS < 1) or neutral evolution (dN/dS = 1) within the genome (Revers et al., 1996; Nielsen & Yang, 1998; Zhou, Gu & Wilke, 2010). These changes can be quantified to determine the rate of evolution of the virus. Rapid evolutionary rates can lead to new viral strains circulating, while slower evolution rates provide stability in the viral population. Virus recombination has a profound effect on the molecular evolution of viruses. Particularly in the *Potyviridae* family, it is a relatively common occurrence within species, and sometimes even between species (Karasev & Gray, 2013; Kehoe et al., 2014a; Hadidi et al., 2016; Blawid, Silva & Nagata, 2017).

Understanding the evolution of viruses is essential in plant breeding efforts due to direct correlation between virus evolution and durability of the resistance within the different cultivars (García-Arenal & McDonald, 2003). This therefore provides a good indicator on the possible duration of each resistant cultivar being developed and released to smallholder farmers. This study therefore sought to answer the following questions:

1 What are the evolutionary patterns of bean common mosaic necrosis virus and cowpea aphid-borne mosaic virus from the western highlands of Kenya?
2 What selective pressures are these viruses under and how do they govern the evolution of these viruses?

To answer these questions, we used a high throughput sequencing method (RNA-Seq) on viral symptomatic beans and cowpeas. NGS applications in plant virology began in 2009 (Blawid, Silva & Nagata, 2017) and have been increasingly applied in the *de novo* discovery of RNA and DNA viruses as well as viroids due to their rise as a rapid and relatively inexpensive mode of viral detection (Wamonje et al., 2017; Ndunguru et al., 2015). Previous reports of using NGS for novel viral discovery and subsequent evolutionary analysis are well-documented (Ndunguru et al., 2015; Kraberger et al., 2017). In this study, we used this approach to obtain near-complete genomes of BCMNV and CABMV and gain further insights into the evolutionary relationships of BCMNV and CABMV.

Bayesian phylogenetic analyses were carried out using the near-complete genomes and also using the individual gene segments. Viral evolution was assessed within sites and across tree branches by determining the selective pressure that these viruses were under. An analysis to determine the likely location of recombination breakpoints along the near-complete genomes from this study and those from GenBank was also carried out. This dataset provides insight into BCMNV and CABMV molecular evolution.

## Materials and Methods

### Field collection

Field surveys were carried out within the highlands of western Kenya over two cropping seasons (2015 and 2016). Fieldwork activities were coordinated through Cassava Diagnostic project Kenya node. Sampling was conducted in heterogeneous cropping systems predominately comprising of beans (*Phaseolus vulgaris L*) (from Busia, Bungoma, Kakamega and Vihiga counties) and cowpea (*Vigna unguiculata (L.) Walp.*) (from Busia county). A total of 120 fields were sampled across the two years during the long rain seasons. Targeted sampling was applied to select fields with mixed cropping systems and in particular cassava and beans. The distance between any two fields was at least five kilometres. In total, we collected beans (*n* = 116) and cowpea (*n* = 10) during the two seasons. Viral symptomatic leaf samples were characterised by leaf chlorosis and mosaic symptoms. Individual leaf samples were stored using two methods: silica gel and the paper press method (Almakarem et al., 2012). Samples were then transported to the Bioscience eastern central Africa (BecA-ILRI) laboratories in Nairobi, Kenya for processing.

### Nucleic acid extraction and PCR screening viruses

From each leaf, RNA was extracted using the Zymo RNA miniprep kit (Zymo, USA) according to the manufacturer’s specifications. The RNA was then lyophilised and shipped to the University of Western Australia for further processing. Screening for positive *Potyvirus* samples was done using the universal *Potyvirus* primers (Webster, 2008). Two standard controls were used, for the positive control RNA from a BCMV positive sample, while the negative non-template control (nuclease free) water was used.

### cDNA library preparation and RNA-Seq sequencing

A total of 28 individual cDNA libraries of each of the samples (*n* = 24 beans, *n* = 4 cowpea) were prepared using Illumina Truseq stranded total RNA sample preparation kit with Plant Ribozero depletion as described by the manufacturer (Illumina). Briefly, ribosomal RNA was depleted followed by viral RNA fragmentation. First strand cDNA was synthesised using random hexamer primers. Subsequently, second strand cDNA was synthesised followed by adapter ligation. Individual samples were used for library preparation, and each sample was dual-indexed with unique barcodes, for identification of the viral genomes recovered. All libraries containing the correct insert size fragment and quantity were sent to Macrogen Korea for subsequent sequencing. Libraries were normalised based on concentration and then pooled before sequencing. Pair-end sequencing (2 × 150 bp) was done on the rapid run mode using a single flow cell on the Illumina Hiseq 2500.

### Assembly and mapping of RNA-Seq reads

Raw reads were trimmed and assembled using CLC Genomics Workbench (CLCGW ver 7.0.5) (Qiagen). Trimmed reads were assembled using the following parameters: quality scores limit set to 0.01, maximum number of ambiguities was set to two and read lengths less than 100 nt were discarded. Contigs were assembled using the *de novo* assembly function on CLCGW with default automatic word size, and automatic bubble size parameters. Minimum contig length was set to 500, mismatch cost two, insertion cost three, deletion cost three, length fraction 0.5 and similarity fraction 0.9. All the contigs were subjected to Blastn and Blastx (NCBI) on the Magnus Supercomputer at Pawsey. Contigs that matched plant viruses were identified and exported to Geneious 8.1.8 (Biomatters). Reference-based mapping was then carried out using complete genomes retrieved from GenBank reference (KX302007) for BCMNV, while for CABMV the closet complete match was MF179118 (this study). Mapping parameters were set as follows: minimum overlap 10%, minimum overlap identity 80%, allow gaps 10% and fine-tuning iteration up to 10 times. The consensus contig from the mapping was aligned using MAFFT (Katoh & Standley, 2013) to the *de novo* contig of interest. The resulting alignments were manually inspected for ambiguities, which were corrected with reference to the original assembly or mapping. The open reading frame and annotation of the final sequences were done in Geneious 8.1.5 (Biomatters). Sequences were referred to as nearly complete if the entire coding region was present, and complete if the entire genome including untranslated regions were present.

### Detection of recombination breakpoints

Assessment of the recombination breakpoints of the nearly complete genomes of BCMNV (*n* = 11) and CABMV (*n* = 2) from this study and those retrieved from GenBank (BCMNV *n* = 9 and CABMNV *n* = 6) was carried out using the seven programs within the RDP4 software (Martin et al., 2015). The programs used were: RDP (Martin et al., 2005), GENECONV (Padidam, Sawyer & Fauquet, 1999), Bootscan (Salminen et al., 1995) MaxChi (Smith, 1992) Chimaera (Posada & Crandall, 2001), 3Seq (Boni, Posada & Feldman, 2007) and SiScan (Gibbs, Armstrong & Gibbs, 2000). A recombination event was detected if found by at least four of the seven programs and further supported by a Bonferroni correction with a *P* value cut-off of 0.05 as described in previous studies (Webster et al., 2007; Kehoe et al., 2014b).

### Bayesian phylogenetic analysis of BCMNV and CABMV

Bayesian inference was used to establish the phylogenetic relationships within BCMNV and CABMV from this study with bean common mosaic virus (BCMV) as the outgroup. Since both the BCMNV and CABMV viruses belong to the *Potyviridae*, a combined phylogenomic tree was constructed. Individual gene trees correlated with the BCMNV and CABMV combined phylogenomic tree. In addition, species-specific genomic trees of BCMNV and CABMV genomic trees were also constructed. The most suitable evolutionary models were determined by jModelTest (Darriba et al., 2012) and bModelTest (Bouckaert & Drummond, 2017). Bayesian analysis of the nearly complete genomes was carried out using Exabayes 1.4.1 (Aberer, Kobert & Stamatakis, 2014) while individual genes were analysed using MrBayes 3.2.2 (Huelsenbenk & Ronquist, 2001). Exabayes was selected for genome analysis since it assigns an independent evolutionary model to each of ten individual genes within a single run. MrBayes was run for 50 million generations on four chains, with trees sampled every 1,000 generations using GTR + I + G as the evolutionary model. In each of the runs, the first 25% (2,500) of the sampled trees were discarded as burn-in. In the ExaBayes run, each gene segment was assigned an independent evolutionary model. ExaBayes was run for 50 million generations on four chains. In each run, the first 25% of the sampled trees were discarded as burn-in. Convergence and mixing of the chains were evaluated using Tracer v1.6 (Rambaut et al., 2014) with effective sample size (ESS >200) indicating convergence. The consensus trees were visualised and annotated using FigTree (http://tree.bio.ed.ac.uk/software/figtree).

### Analysis of extent of selection pressure across sites with whole genome

The extent of selective pressure was measured by computing dN/dS across sites of the coding region using SNAP (https://www.hiv.lanl.gov/content/sequence/SNAP/SNAP.html).

We further identified sites under positive selection using SLAC in Datamonkey (http://www.datamonkey.org/).

### Analysis of selection pressure

Selection pressure was analyses of BCMNV and CABMV was done using a maximum likelihood codon-substitution model in the CODEML program in PAML 4 (Yang, 2007). A representative subset (*n* = 14; *n* = 10 from this study and *n* = 4 representative from GenBank) from the phylogenomic tree obtained above was used in the analysis. Two comparisons between the models were made and assessed by a likelihood ratio test (LRT): (i) M2a vs M1a (neutral vs positive selection) (ii) M3 vs M0 (variable ratio vs one single ratio) with significance level *α* = 0.05.

### Testing for selection pressure between clades of the gene trees

Differences in the selection pressure between the two main clades I and II of BCMNV were assessed by testing the null hypothesis of equal selection pressure (Model = 0 Nsite = 0) against the alternative hypothesis that each clade has a distinct *ω* (Model = 1 Nsite = 0, different selection pressure) across all ten gene trees. These hypotheses were evaluated using the likelihood ratio test (LRT) with significance level *α* = 0.05.

## Results

### Sample screening next-generation sequencing and data assembly

Samples that were negative after PCR amplification were excluded from library preparation. RNA-Seq on total plant RNA after trimming and assembly ranged between 12,667,976 and 15,638,762 reads. *De novo* assembly produced a number of contigs ranging between 895-19,029 nucleotides (nt) (Table S2). Plant virus contigs were identified after BLASTn searches with lengths of between 1,048–9,763 nt, and average coverage depth of between 71 and 8,924 times. Genomes with the complete open reading frames and including the complete untranslated regions were considered to be full genomes. However, genome sequences that lacked parts of the 5′ and 3′ UTR regions were referred to as near-complete genomes. The final sequence was obtained from the consensus of *de novo* assembly and the mapped consensus of reads and ranged from 8,241 to 9,837 nt in length (Table S2). BCMNV and CABMV sequences obtained from this study are summarised in Table 1, while sequences retrieved from GenBank and associated metadata are provided in Table S1. In total, there were 11 BCMNV and two CABMV genomes from this study (Table 1). All viral sequences generated from this study were deposited in GenBank with the accession numbers MF179108–MF179120. In addition, raw reads were deposited in the NCBI Short read Archive under the following accession numbers SRR7896282–SRR7896271.

**Table 1:** Near complete genomes of *Bean common mosaic necrosis virus* (BCMNV) and the *Cowpea aphid-borne mosaic virus* (CABMV) sampled over two seasons 2015/2016 in the Western highlands of Kenya.

Sample ID	GenBank accession number	Virus identified	Host plant	Geographical location	Year of sampling
SRF 08	MF179117	BCMNV	Bean	Kakamega	2015
SRF 33	MF179112	BCMNV	Bean	Kakamega	2015
SRF 50	MF179115	BCMNV	Bean	Kakamega	2016
SRF61	MF179109	BCMNV	Bean	Busia	2016
SRF 74	MF179120	CABMV	Cowpea	Bungoma	2016
SRF 75	MF179111	BCMNV	Bean	Bungoma	2016
SRF 77	MF179118	CABMV	Cowpea	Bungoma	2016
SRF 97	MF179113	BCMNV	Bean	Kakamega	2016
SRF 99	MF179110	BCMNV	Bean	Kakamega	2016
SRF111	MF179119	BCMNV	Bean	Maseno	2016
SRF 114	MF179116	BCMNV	Bean	Vihiga	2016
SRF 119	MF179114	BCMNV	Bean	Vihiga	2016
SRF 122	MF179108	BCMNV	Bean	Vihiga	2016

### Analysis of recombination in the genomes of BCMNV and CABMV

Two BCMNV isolates from Kenya (Table 2) and three CABMV isolates (Table 3) from Brazil and India were identified as recombinant sequences. The locations of recombination were within the P1/HcPro/P3 in (BCMNV) and Nla Pro/Nlb/CP regions in (CABMV) (Tables 2 and 3).

**Table 2:** Recombination signals across *Bean common mosaic necrosis virus* (BCMNV) using RDP4. Table entries represent the recombinant sequences and the position of recombination breakpoints in the near complete genomes. A recombination pattern was considered if supported by at least four of the seven RDP4 programs at a significance level of 0.05.

Recombination events	Recombinant sequence	Detected breakpoint	Parental sequence (Major)	Parental sequence (Minor)	Detected in RDP4	Avr *P*-Val
1	SRF08_MF179117	5515-8886	Kenya_Beans_SRF50	USA_Beans_HQ229994	**R**, M, C, S, 3	3.55 × 10^−4^
2	SRF114_MF179116	1341-1619	Kenya_Beans_SRF50	USA_Beans_HQ229993	R, G, B, C **M** 3	0.0415

**Notes.**

Key: Recombinant programs in RDP4 that detected recombinant events across the near complete genome of BCMNV.

3 3seq B Bootscan C Chimera G Gencov R RDP M Maxchi S Siscan

The bold letters in the column (detected in RDP4) indicate the program with the highest *p*-value.

Avr *P*-Val average binomial *P*-value

**Table 3:** Recombination signals across *Cowpea aphid-borne mosaic virus* (CABMV) using RDP4. Table entries represent the recombinant sequences and the position of recombination breakpoints in the near complete genomes. A recombination pattern was considered if supported by at least four of the seven RDP4 programs at a significance level of 0.05.

Recombination event	Recombinant sequence	Detected breakpoint	Parental sequence (Major)	Parental sequence (Minor)	Detected in RDP4	Aver *P* Value
1	Brazil_ HQ880243	2,332–2,396	Zimbabwe_ NC004013.1	Brazil_Cowpea_ HQ880242	R, G, B, M, C, S,**3**	2.442 × 10^−14^
2	India_ KM597165	917–1,220	Brazil_ HQ880242	Brazil_Cowpea_ HQ880243	R, G, B, M, C, **S**, 3	1.800 × 10^−14^
3	Brazil_ HQ880242	1,385–1,409	India_ KM597165	Brazil_Cowpea_ HQ880243	R, G, B, M, C, **S, 3**	1.571 × 10^−07^
6	Brazil_ HQ880243	8,643–8,809	India_ KM597165	Uganda_Cowpea_ KT726938	R, G, B, **M**, C, 3	1.328 × 10^−04^
7	Brazil_ HQ880242	94–275	Zimbabwe_ NC004013.1	Uganda_Cowpea_ KT726938	R, **G**, B, M, C, 3	4.750 × 10^−4^
	India_ KM597165					
10	Brazil_ HQ880243	6,605–8,134	Zimbabwe_ NC004013.1	Brazil_Cowpea_ HQ880242	R, B, M, **C**, S	2.595 × 10^−2^
18	Brazil_ HQ880243	6,916–8,065	Uganda_ KT726938	Zimbabwe_Cowpea_ NC004013.1	R, **B**, M, C, S	1.664 × 10^−2^
	Brazil_ HQ880242					
	India_ KM597165					

**Notes.**

Key: Recombinant programs in RDP4 that detected recombinant events across the near complete genome of CABMV.

3 3seq B Bootscan C Chimera G Gencov R RDP M Maxchi S Siscan

The bold letters in the column (detected in RDP4) indicate the program with the highest *p*-value.

### Bayesian evolutionary relationship of BCMNV and CABMV

**Figure 1:**
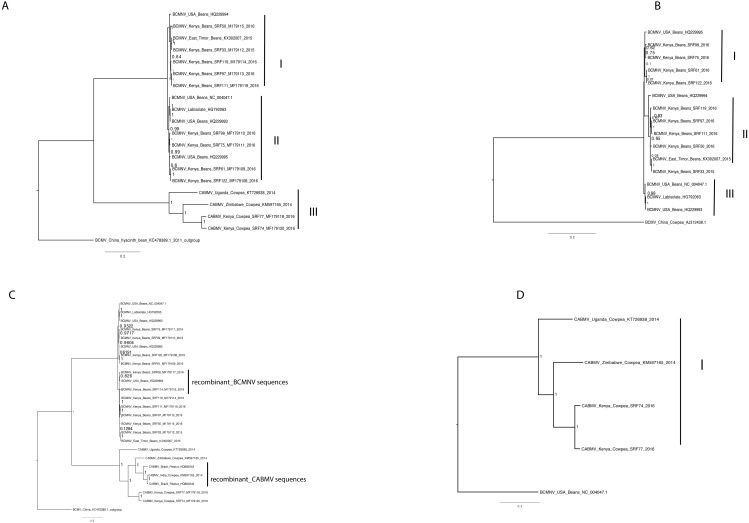
Bayesian analysis of near complete genome of *Bean common mosaic necrosis virus* (BCMNV) and *Cowpea aphid-borne mosaic virus* (CABMV) with nodes in each branch labeled with posterior probabilities. *Bean common mosaic virus* (BCMV) was used as the out group to root the tree (B) Bayesian analysis of near complete genome of *Bean common mosaic necrosis virus* (BCMNV) with nodes in each branch labeled with posterior probabilities. (C) Bayesian analysis of near complete genome of *Cowpea aphid-borne mosaic virus* (CABMV) with nodes in each branch labeled with posterior probabilities. The scale indicates the nucleotides substitutions per every 100 sites across the near complete genome trees. Tip labels contains information on: virus, country of sample collection, isolation source, GenBank and/or field identification and year of sampling.

**Figure 2:**
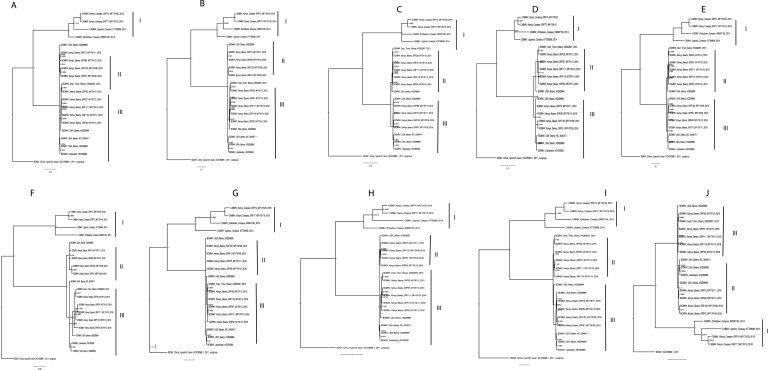
Consensus trees sampled in a Bayesian analysis of *Bean common mosaic necrosis virus* (BCMNV) and *Cowpea aphid-borne mosaic virus* (CABMV) (A), coat protein (CP) (B) CI (C) Nlb (D) PI (E) Hc-Pro (F) P3 (G) Nla-Pro (H) Nla-Vpg (I) 6K2 (J) 6K1. The scale bar represents the nucleotide substitution per every 100 sites within each gene. Tip labels contain information on: virus, country of sample collection, isolation source, GenBank and/or field identification and year of sampling.

Phylogenetic relationships within BCMNV and CABMV were based on the near-complete genome tree (Figs. 1A, 1B and 1C) and with reference to the ten individual gene trees. Both the whole genome tree and the individual gene trees gave similar results. They identified two well-supported clades, identified as I and II, within BCMNV and one clade within CABMV, identified as clade III (Fig. 1A). Species-specific genome trees resolved three main clades identified as I–III within BCMNV (Fig. 1B) while the CABMV genome tree resolved a single monophyletic clade identified as I (Fig. 1C). The individual gene trees resolved similar clades (I–III) to the combined BCMNV-CABMV phylogenetic tree (Fig. 2). Across the two BCMNV clades (I–II) the percentage nucleotide identities were 96.1–98.9% as would be expected for members of the same *Potyvirus* species. Likewise, the CABMV clade (III) showed 79.3% nucleotide identity, as expected for within species sequences, but showing higher within-species diversity compared to the BCMNV sequences (Table 4).

**Table 4:** Pairwise sequence comparisons of the nearly complete genomes of representative sequences across the *Bean common mosaic necrosis virus* (BCMNV), *Cowpea aphid-borne mosaic virus* (CABMV) with Bean common mosaic virus ( KC478389.1) as the outgroup using Geneious 8.1.8. Intraclade similarity was over 79% across all the main clades.

	SRF77_ MF179118_ BCMNV	CABMV_ KT726938_ BCMNV	SRF99_ MF179110_ BCMNV	SRF75_ MF179111_ BCMNV	SRF33_ MF179112_ BCMNV	SRF119 MF179114_ BCMNV	BCMV_ KC478389.1
SRF77_MF179118_BCMNV							
CABMV_KT726938	79.3						
SRF99_MF179110_BCMNV	66.9	67.0					
SRF75_MF179111_BCMNV	66.9	66.9	99.1				
SRF33_MF179112_BCMNV	66.9	66.6	96.2	96.3			
SRF119_MF179114_BCMNV	66.9	66.6	96.1	96.2	98.9		
BCMV_KC478389.1	64.2	64.9	66.3	66.3	66.4	66.3	

**Notes.**

Clade III 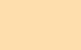

Clade II 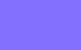

Clade I 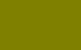

CABMV Cowpea aphid-borne mosaic virus BCMNV Bean common mosaic necrosis virus BCMV Bean common mosaic virus

### Estimation of selection pressure across sites and branches of the whole genome

**Figure 3:**
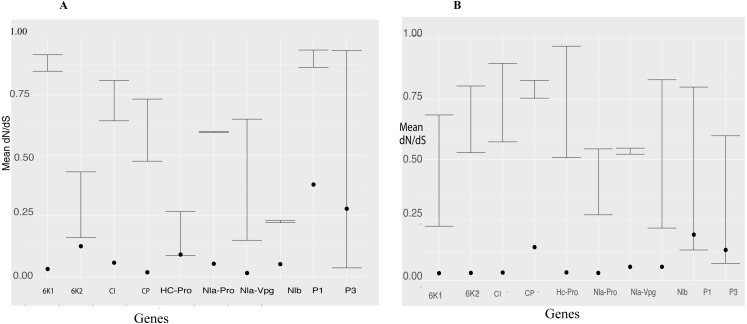
Selection pressure within sites across the viral gene fragments was determined by assessing the average synonymous and non-synonymous (dN/dS) across the coding region using SNAP and SLAC that were plotted against each gene. Confidence interval of the dN/dS ratio are provided. All sites are under strong purifying selection with dN/dS values < 1 (A) Comparison of the average synonymous (dS) and non-synonymous (dN) sites across the coding region of each gene in bean common mosaic necrosis virus (BCMNV) near complete genome using SNAP. (B) Comparison of the average synonymous (dS) and non-synonymous (dN) across the coding region of each gene cowpea aphid-borne mosaic necrosis virus (CABMV) near complete genome using SNAP. The dots on the graph represent the average dN/dS in each codon of the gene fragment.

We used the branch model in CODEML within PAML 4 to estimate episodic positive selection pressure along the branches of BCMNV, BCMV and CABMV phylogenetic tree (Fig. 2). Model 2 indicated independent episodic changes along the branches of BCMNV, CABMV and BCMV was rejected based on a *p*-value of >0.05 (Table 5); thus supporting the M_0_ of equal episodic changes within BCMNV, CABMV and BCMV (Table 5). In addition, the selective pressure within the genes of both BCMNV and CABMV supported high purifying selection *ω* <1 (Fig. 3) across all genes. However, the site-selective pressure as determined by analysis using SNAP was not uniform across the genes (Fig. 3). Selective pressure within BCMNV genes was highest in Nlb, Nla-Vpg and lowest in PI and P3 (Fig. 3A). In CABMV genes, the highest site-selective pressure site were 6K1 and Nla-Pro, while the least site pressure was in P1 and P3 (Fig. 3B). The two clades within BCMNV gene trees were under equal selective pressure except within the CI gene (Table 6).

**Table 5:** The dN/dS (ω) values, log-likelihood (lnL) values, likelihood ratio test (LRT) statistics and positively selected branches under different models of codon substitution were used to investigate selection pressures on 8883 nucleotides of *Bean common mosaic necrosis virus* (BCMNV), *Bean common mosaic virus* (BCMV) and *Cowpea aphid-borne mosaic virus* (CABMV).

Model	Background	Foreground	Parameter estimate	^*^Degrees of freedom	lnL	^**^LRT
M0	*ω*_**0**_= BCMNV = CABMV	–	*ω*_1_ = 0.03287	–	−37878.264515	–
M2	*ω*_**0**_= BCMNV	*ω*_**1**_= CABMV	ω_0_= 0.05721, ω_1_= 0.05129	2	−34294.217766	7168.093498^***^
M2	*ω*_**0**_= CABMV	*ω*_**1**_= BCMNV	ω_0_= 0.05712, ω_1_= 0.05084	2	−34294.206420	7168.11619^***^
M1	–	–	ω= 0.00010-range	27	−34210.774645	7334.979738^***^

**Notes.**

* Degrees of freedom used for the LRT, computed as the difference in the number of parameters under the null and alternative hypotheses.

** Likelihood ratio test (LRT) statistic, calculated as 2*(lnLx ( lnL0)).

*** Indicates significance at level *α* = 0.05.

**Table 6:** Comparison of rates of evolution within the two clades within *Bean common mosaic necrosis virus* (BCMNV) across the different genes. The null hypothesis is that there are equal rates while the alternative specifies different ω values between the two clades. CI tests were significant at the 0.05 levels.

	Equal rates (null hypothesis)		Differing rates (alternative hypothesis)				
Gene	lnL	ω (d_*N*_/d_*S*_)	lnL	ω0	ω1	LRT statistic	*P* value
P1	−2214.505595	0.37095	−2214.452326	0.37610	0.30129	0.106538	0.7441205
HcPro	−2795.992899	0.09728	−2794.811926	0.08884	0.60647	2.361956	0.1243262
P3	−2055.983286	0.28547	−2054.343977	0.27297	999.00000^***^	3.27862	0.07018792
6K1	−323.414987	0.05870	−323.205094	0.06221	0.00010^***^	0.419786	0.5170438
CI	−3606.604936	0.08320	−3602.434146	0.06552	0.32492	8.341508	0.003874942
6K2	−341.075396	0.24049	−341.075137	0.24140	0.23425	0.00052	0.981807
Nla-Vpg	−979.236913	0.09398	−978.201866	0.11081	0.00010^***^	2.07009	0.1502127
Nla-Pro	−1392.256063	0.08420	−1391.975219	0.08673	0.00010^***^	0.56168	0.4535841
Nlb	−2997.347051	0.06322	−2997.340969	0.06285	0.07124	0.012164	0.912179
CP	−735.455148	0.09977	−735.394608	0.10664	0.07166	0.12108	0.7278661

**Notes.**

*** ω estimates at the boundary of allowable values in PAML.

## Discussion

Legumes play a critical role in providing food and nutritional security. However, viral diseases severely affect their yields. This study provides insights into the phylogenomic relationships of BCMNV and CABMV and selective pressures that govern their evolution. We present the near-complete genomes of eleven BCMNV and two whole genomes of CABMV from Kenya. These viruses were under strong purifying selection with BCMNV and CABMV evolving independently but at a slow rate. These genomes are the first from SSA and are an invaluable genomic resource to help understand the molecular evolution of these viruses and for the development of molecular diagnostic tools.

### Recombination and phylogenomic analysis of BCMNV and CABMV

One of the drivers of viral evolution is recombination (Roossinck, 2003; Valli, López-Moya & García, 2007). In this study, we identified recombinant sequences in BCMNV (Table 2) and in CABMV (Table 3). Previous reports have indicated that BCMV-BCMNV recombination occurs resulting in new, stable and virulent BCMNV strains (Larsen et al., 2005). Similar findings have been reported in other RNA viruses such as turnip mosaic virus (Nguyen et al., 2013), papaya ringspot virus (Maina et al., 2017) and cassava brown streak virus (Ndunguru et al., 2015). Since recombinant sequences can distort the true relationships when studying the phylogenetic relationships between sequences, we excluded all recombinants from the subsequent downstream analysis (Varsani et al., 2008; Penny et al., 2008). Moreover, Wainaina et al., 2018 reported the effects of recombination on the phylogenomic trees, which was also observed in this study (Fig. 1C). Whole genomes and the nearly complete genome of BCMNV and CABMV were used to determine the phylogenomic relationships within these two *Potyviruses*. We identified three well-supported clades in BCMNV and one clade in CABMV within the phylogenomic trees (Figs. 1A–1C). We suspect that the drivers of BCMNV and CABMV diversity are similar to other members of the *Potyviridae*. Within *Potyviridae* the evolutionary divergence is thought to occur through the starburst phenomena, that happens during the introduction of new viruses onto native lands (Gibbs et al., 2008). Native ecosystems act as catalysts for genetic divergence of these viruses resulting in new viral strains or quasispecies. The similarity in tree topologies between the genome tree and the coat protein gene tree indicate the coat protein is a reliable phylogenetic marker (Figs. 1A–1C and 2A) and the topologies recovered in our analyses are similar to previously reported topologies (Zheng et al., 2002). However, our results differ from previous studies within some members of *Potyviridae* (cassava brown streak virus, Uganda cassava brown streak virus), where the whole genome tree and their coat protein gene tree did not concur with the species trees (Alicai et al., 2016). This highlights the diversity of the members of the *Potyviridae* and the importance of performing rigorous phylogenetic, recombination and evolutionary analysis for each species*.* However, it is worth noting that this difference could be due to Alicai et al. (2016) comparing the coalescent species tree with the respective gene trees, which was not the case in this study.

### Selective pressure analysis across phylogenetic tree branches

We identified equal episodic selection pressure on the BCMNV, CABMV and BCMV branches of the phylogenomic tree (Table 5). However, each of the clades within the gene trees of BCMNV was under equal selection pressure (Table 6) except for the CI gene tree, while coding sites of both BCMNV and CABMV were under strong purifying selection (Fig. 3), in particular, within Nlb, Nla-Vpg in BCMNV and 6K1 and Nla-Pro in CABMV (Fig. 3). These genes are associated with viral genome replication. The continuous survival of viruses is dependent on successful replication, which could be a key driver in ensuring that these genes undergo high purifying selection pressure in spite of recombination and mutations that may occur as observed (Tables 2 and 3) to ensure they maintain these critical functions. These findings are similar to other vector-transmitted RNA viruses such as the *Rice stripe virus* (RSV) (Wei et al., 2008; He, Guan & He, 2017). A slower rate in the evolution of BCMNV and CABMV could be beneficial to breeding efforts since it would allow for the new BCMNV resistant varieties under development to persist for longer than they would if the viruses were evolving at faster rates. The importance of continuous monitoring of viral evolution ensures that breeding efforts against viruses remain relevant. This is especially important considering the numerous cases of resistance breakdown associated with continuous virus evolution. There is a direct correlation between the evolution of viruses and the durability of the resistance (García-Arenal & McDonald, 2003). This is especially the case within *Potyviridae* where recombination is a frequent driver of new viral strains.

### Limitations of next generation sequencing in viral diagnosis

Viral diagnosis using NGS has multiple advantages that allow for the detection of viruses even at low titres, which has subsequently resulted in the increased identification of viral diversity (Simmonds et al., 2017). This approach is faced with several limitations that include chimera formation during *de novo* assembly, difficulty in the accurate assembly of segmented virus as well as assembling endogenous viruses that are may be integrated within host. Practices such as confirmation by direct Sanger sequencing and follow up with biological infectivity assays will further confirm the results of these types of experiments, including those conducted in this study. In addition, this study provides a snapshot on the bean and cowpea viruses within the western highlands of Kenya. More extensive sampling could uncover higher viral diversity in beans and cowpea.

## Conclusions and Future Perspectives

In this study, we identified two main clades within BCMNV and a single clade within CABMV based on phylogenomic analysis using the whole genome and ten gene trees. The overall evolutionary rates within BCMNV and CABMV revealed the viruses were under strong purifying selection and thus are evolving slowly. These findings provide robust genomic and evolutionary data to complement current bean breeding efforts underway in Africa. In addition, this study highlights the need to establish robust biosecurity and phytosanitary measures within developing countries to control the spread of these viruses. That they appear to be relatively stable in an evolutionary sense at this point bodes well for the development of plant disease management strategies.

##  Supplemental Information

10.7717/peerj.6297/supp-1 Table S1 Supplemental Table 1 Summary of GenBank genomic sequences of *Bean common mosaic necrosis virus* (BCMNV) and *Cowpea aphid-borne mosaic virus* (CABMV) used in this study for Bayesian phylogenetic analysis.

10.7717/peerj.6297/supp-2 Table S2 Supplemental Table 2 *Denovo* assembly and mapping of *Bean common mosaic necrosis virus* (BCMNV) and *Cowpea aphid-borne mosaic virus* (CABMV) reads using CLC Genomic Workbench version 8.0.5 and Geneious version 8.1.8 from bean and cowpea samples sequenced from the Western highlands of Kenya.

## Additional Information and Declarations

### Data Availability

The following information was supplied regarding data availability: GenBank: MF179108–MF179120.
