# Supplementary material for: Evolutionary insights of Bean common mosaic necrosis virus and Cowpea aphid-borne mosaic virus
Source: PeerJ. 2019 Feb 13;7:e6297. doi: 10.7717/peerj.6297 (PMC6377593; doi:10.7717/peerj.6297)
Supplement: Table S1 — Summary of GenBank genomic sequences of Bean common mosaic necrosis virus (BCMNV) and Cowpea aphid-borne mosaic virus (CABMV) used in this study for Bayesian phylogenetic analysis. [file peerj-07-6297-s001.docx]

| Accession | Country | Host | Virus | Sequencing Platform | Region |
| --- | --- | --- | --- | --- | --- |
| KX302007 | East Timor | Beans | BCMNV | Hiseq Illumina | Genome |
| HG792063 | Lab Isolate | Beans | BCMNV | Not recorded | Genome |
| HQ229993 | USA | Beans | BCMNV | Not recorded | Genome |
| HQ229994 | USA | Beans | BCMNV | Sanger | Genome |
| HQ229995 | USA | Beans | BCMNV | Sanger | Genome |
| NC_004047.1 | USA | Beans | BCMNV | Sanger | Genome |
| KT726938 | Uganda | Cowpea | CABMV | Miseq | Genome |
| KM597165 | Zimbabwe | Cowpea | CABMV | Sanger | Genome |
