# Supplementary material for: Evolutionary insights of Bean common mosaic necrosis virus and Cowpea aphid-borne mosaic virus
Source: PeerJ. 2019 Feb 13;7:e6297. doi: 10.7717/peerj.6297 (PMC6377593; doi:10.7717/peerj.6297)
Supplement: Table S2 — Denovo assembly and mapping of Bean common mosaic necrosis virus (BCMNV) and Cowpea aphid-borne mosaic virus (CABMV) reads using CLC Genomic Workbench version 8.0.5 and Geneious version 8.1.8 from bean and cowpea samples sequenced from the Western highlands of Kenya. [file peerj-07-6297-s002.docx]

| Sample ID | GenBank  Accession | Virus | No.of reads | No of reads after trimming | Number of contigs produced | Ref seq used for mapping | Length of consensus sequence from mapping (Geneious)* | No. Reads mapped to Ref.sequence | Mean coverage (Geneious) | Contig positive for virus and length | Average coverage (CLCGW) | Number of reads mapped to contigs of interest | % Similarity BLAST | Final sequence length | No of Ambiguities |
| --- | --- | --- | --- | --- | --- | --- | --- | --- | --- | --- | --- | --- | --- | --- | --- |
| SRF 50 | MF179115 | BCMNV | 14,530,332 | 12,682,044 | 9381 | KX302007 | 9592 | 263543 | 4140 | 10 (9592) | 4721 | 351905 | 98 | 9592 | None |
| SRF61 | MF179109 | BCMNV | 15,229,420 | 13,796,054 | 17,253 | KX302007 | 10,085 | 572,352 | 8924.1 | 10(9652) | 9240 | 12 | 99 | 9632 | None |
| SRF 97 | MF179113 | BCMNV | 15,040,964 | 13,307,406 | 7264 | KX302007 | 9327 | 2311866 | 36120.50 | 4(3189), 11(1,945), 22(1,452), 33 (1,048) | 45978;  32642;  29,872;  33425 | 1121588; 492372;  468611;  273456 | 98;  98;  98;  98 | 9327 | None |
| SRF 08 | MF179117 | BCMNV | 12879188 | 8,883,986 | 1800 | KX302007 | 10846 | 1306333 | 18245.2 | 1(6550)  2(3257) | 2,004,  518,808 | 1017797;  233770 | 98; 97 | 9639 | None |
| SRF 33 | MF179112 | BCMNV | 12,633,204 | 8,550,351 | 895 | KX302007 | 9870 | 2,758,857 | 419164.4 | 2(1,623)  3(1,616);  4(5720) | 9201;  32492  43645 | 1126551;  850522 | 98 | 9870 | None |
| SRF 99 | MF179110 | BCMNV | 12,123,604 | 10,545,462 | 3496 | KX302007 | 9900 | 778,994 | 12490.2 | 3(9628) | 14624 | 1,131,939 | 99 | 9900 | None |
| SRF 111 | MF179119 | BCMNV | 14,136,462 | 12,256,865 | 3330 | KX302007 | 9830 | 4604 | 71 | 275(591), 276(1485);  352(1,370);  381(1,018);  433(970);  531(704);  830(1247);1129(1,211) | 71.3; 87.3;  83.15,  45.24;  475;  68.48;  119.08 | 839; 966; 661;  352; 475; 783;  1496 | 97 | 9830 | None |
| SRF 114 | MF179116 | BCMNV | 15,341,998 | 13,780,642 | 17,276 | KX302007 | 10,438 | 1121412 | 16792.8 | 9(7973);47 (1689) | 19048; 14,151 | 1,135105;  182,702 | 98; 97 | 9631 | None |
| Sample ID | **GenBank**  **Accession** | **Virus** | **No.of reads** | **No of reads after trimming** | **Number of contigs produced** | **Ref seq used for mapping** | **Length of consensus sequence from mapping (Geneious)*** | **No. Reads mapped to Ref.sequence** | **Mean coverage (Geneious)** | **Contig positive for virus and length** | **Average coverage (CLCGW)** | **Number of reads mapped to contig of interest** | **% Similarity BLAST** | **Final sequence length** | **No of Ambiguities** |
| SRF 119 | MF179114 | BCMNV | 10,842,066 | 9,449,543 | 3057 | KX302007 | 9895 | 504,667 | 7957.3 | 2(9672) | 91458 | 712,676 | 98 | 9895 | None |
| SRF 122 | MF179108 | BCMNV | 15,638,762 | 14,128,655 | 19,029 | KX302007 | 9837 | 36,737 | 563.8 | 472(9624) | 559.6 | 38545 | 99 | 9837 | Non |
| SRF 75 | MF179111 | BCMNV | 12,677,976 | 11,337,046 | 8802 | KX302007 | 9832 | 53,001 | 819 | 5(5758);  12 (3432);  6233(666) | 49370;  46209;  1940 | 2105916;  1187041;  14889 | 97;98 | 9832 | None |
| SRF 74 | MF179120 | CABMV | 12,933,786 | 11,664,027 | 13,369 | - |  |  |  | 102(9763) | 338.73 | 25,046 | 83 | 9763 | None |
| SRF 77 | MF179118 | CABMV | 13,655,176 | 12,340,439 | 16,251 |  |  |  |  | 5(8,241) | 2153 | 1,302,085 | 84 | 8241 | None |
